# Abdominal pregnancy: a case report and review of 17 cases

**DOI:** 10.1007/s00404-022-06570-9

**Published:** 2022-04-26

**Authors:** Yu Chen, Ping Peng, Chunying Li, Lirong Teng, Xinyan Liu, Juntao Liu, Dongyan Cao, Lan Zhu, Jinghe Lang

**Affiliations:** 1 Department of Obstetrics and Gynecology, Peking Union Medical College Hospital, Chinese Academy of Medical Science and Peking Union Medical College, Beijing, 100010 China; 2 National Clinical Research Center for Obstetrics and Gynecologic Diseases, Beijing, 100010 China

**Keywords:** Abdominal pregnancy, Magnetic resonance imaging, Angiography, Case review

## Abstract

**Purpose:**

To analyze the clinical characteristics of abdominal pregnancy, and to explore the diagnosis and prognosis of different treatment methods.

**Methods:**

The cases of patients with abdominal pregnancy admitted to Peking Union Medical College Hospital between January 1, 1989 and January 1, 2021, were analyzed retrospectively.

**Results:**

The median age of 17 patients was 34 years (22–42 years); the median gestational duration was 57 days (from 41 days to 32 weeks). Among all 17 patients, 15 (88.24%) presented with abdominal pain. The implantation sites of the gestational sac included the bladder peritoneal reflection, anterior wall of the rectum, omentum, serous membrane of the uterus, and inside or on the surface of uterosacral ligament. In all, only 29.41% cases (5/17) were diagnosed before surgery. All 17 patients were treated via surgery. Further, 58.82% (10/17) patients recovered without complications, 29.41% (5/17) developed fever, 5.88% (1/17) underwent reoperation because of intra-abdominal bleeding, and 5.88% (1/17) developed double lower limb venous thrombosis. All 17 patients survived.

**Conclusion:**

The preoperative diagnosis rate of abdominal pregnancy is low. Planting sites in the pelvic peritoneum and pelvic organs are more common than the others. Laparoscopic surgery in the first trimester of pregnancy can achieve better therapeutic effects. However, the blood supply of the placenta should be fully evaluated before surgery. When it is expected that attempts to remove the placenta will cause fatal bleeding, the placenta can be left in place, but long-term close follow-up should be paid attention to.

## Introduction

As a pathological pregnancy, ectopic pregnancies accounts for approximately 1–2% of all pregnancies [1]. Among them, in more than 90%, the implantation site is in the fallopian tube. In abdominal pregnancies, the gestational sac is implanted in the peritoneal cavity outside the uterine cavity or fallopian tube; these cases account for approximately 1.4% of all ectopic pregnancies [2]. The implantation sites in abdominal pregnancy in previous reports have included the following: the omentum, peritoneum of pelvic and abdominal cavity, uterine surface and abdominal organs such as spleen, intestine, liver, large blood vessels in the abdominal cavity, diaphragm, and others [3]. The symptoms and signs in patients vary according to the implantation site. If the implantation site is in the pelvic cavity, early diagnosis is easily confused with tubal ectopic pregnancy [4], and only 20–40% of cases are diagnosed before surgery [5]. Advanced abdominal pregnancy (AAP), that is, an abdominal pregnancy after 20 weeks of gestation, caused by the implantation of an abnormal placenta, can cause severe maternal postpartum hemorrhage and coagulopathy, which could lead to death in severe cases [6]. Accordingly, maternal mortality rate is approximately seven times higher in abdominal pregnancies compared with that in other ectopic pregnancies [7].

In this article, we report a case of abdominal pregnancy at 3 months. The pregnancy was thought to have been terminated at a local hospital during the first trimester, but an abdominal pregnancy was soon after discovered in the second trimester. We share the medical history, imaging findings, diagnosis, and treatment of the patient, and reviewed and summarized the characteristics, diagnosis, and treatment of 17 cases, including the current case, of abdominal pregnancy at our hospital's obstetric center in the past 35 years, hoping to provide a new basis for abdominal pregnancy management.

## Case report

A 39-year-old woman, G7P3, with 3 healthy children. Her last delivery was in 2013. Approximately 45 days ago, an ultrasound at a local clinic revealed a 2-month intrauterine pregnancy, and a medical abortion was conducted subsequently on patient’s request. This was followed with uterine curettage because of abortion failure. The medical records of the patient could not be traced back. After the operation, the patient had vaginal spotting for 5 days, and no more vaginal bleeding or menstrual cramps at the time of consultation. One month after the operation, the patient presented with palpitation and nausea. The local hospital’s ultrasound displayed that the uterus was 8.4 × 6.4 × 6.1 cm large, and the gestational sac was 11.2 × 10.5 × 8.1 cm, and was cited on the left posterior uterus. The fetus was visible inside; the double parietal diameter was 3.2 cm, and the amniotic fluid depth was 3.3 cm, indicating an abdominal pregnancy. Therefore, the patient was transferred to our hospital.

**Fig. 1:**
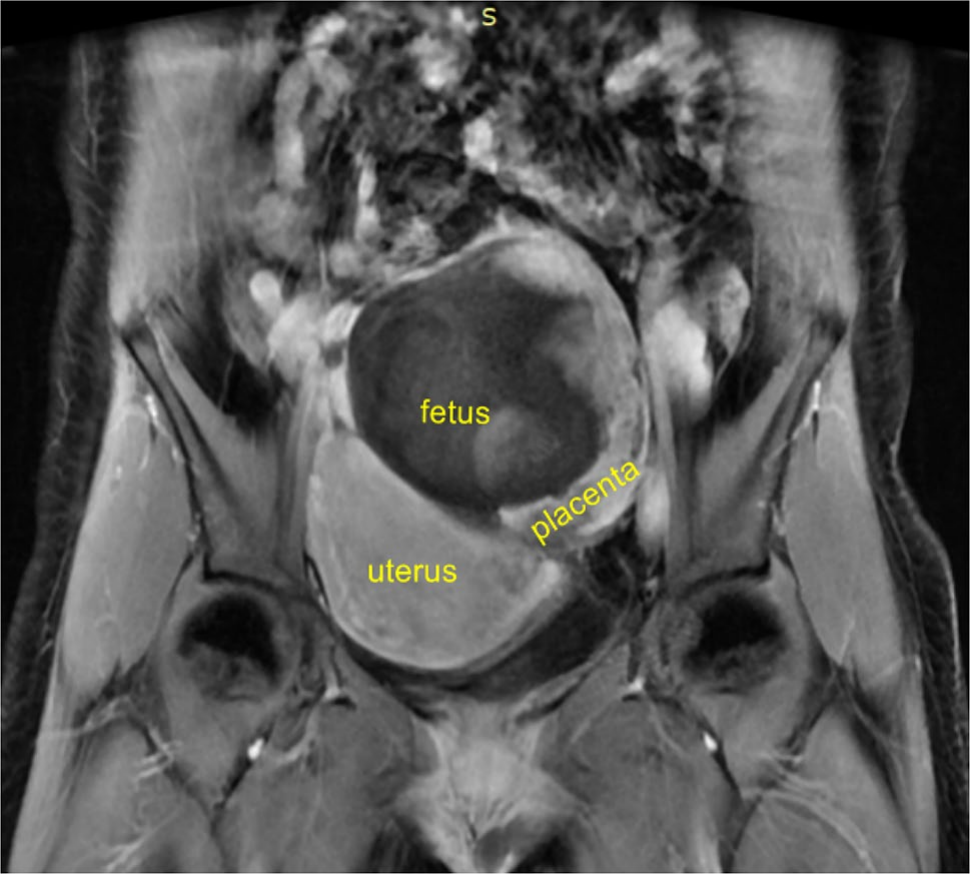
Abdominal pregnancy: MRI coronal position. The fetus is located at the left rear side of the uterus

The patient’s body temperature was 37.5 °C when admitted to our hospital, and she had intermittent mid-abdominal pain. No lower abdominal pain or swelling or vaginal bleeding was observed. Laboratory examinations revealed that the white blood cells were in the normal range, but the proportion of neutrophils had increased to 84.6%, accompanied by an increase in hsCRP of 31.83 mg/L, as well as mild hypokalemia, hypoproteinemia, and mild anemia. Further, β-HCG level was 55,264.9 IU/L. An ultrasound revealed that the uterus was enlarged, the endometrial thickness was 0.8 cm, and the gestational sac was located on the left rear side of the uterus. A formed fetus was seen inside, with a fetal BPD of 3.6 cm, fetal abdominal circumference of 10.6 cm, and fetal femur length of 2.2 cm. The fetal heartbeat was visible, and amniotic fluid depth was approximately 4.0 cm. The placenta was located in the right front of the left iliac blood vessel, with a thickness of approximately 3.5 cm and a length of approximately 16.1 cm. There was no obvious free liquid in the pelvis. The diagnosis was confirmed after magnetic resonance imaging (MRI) (Fig. 1). On MRI, the posterior, right, and anterior pelvic short T1 signals around the placenta were considered as bloody effusions. Vascular ultrasound and angiography confirmed that the blood supply to the placenta came from the left uterine artery and left ovarian artery. Gelatinous sponge particles were used to embolize the left uterine artery and left ovarian artery, and the embolization was smoothly conducted. After 1 h, ultrasound imaging indicated that the fetal heart had stopped beating. Except for slight abdominal pain, there were no other discomforts to the patient later that night.

**Fig. 2:**
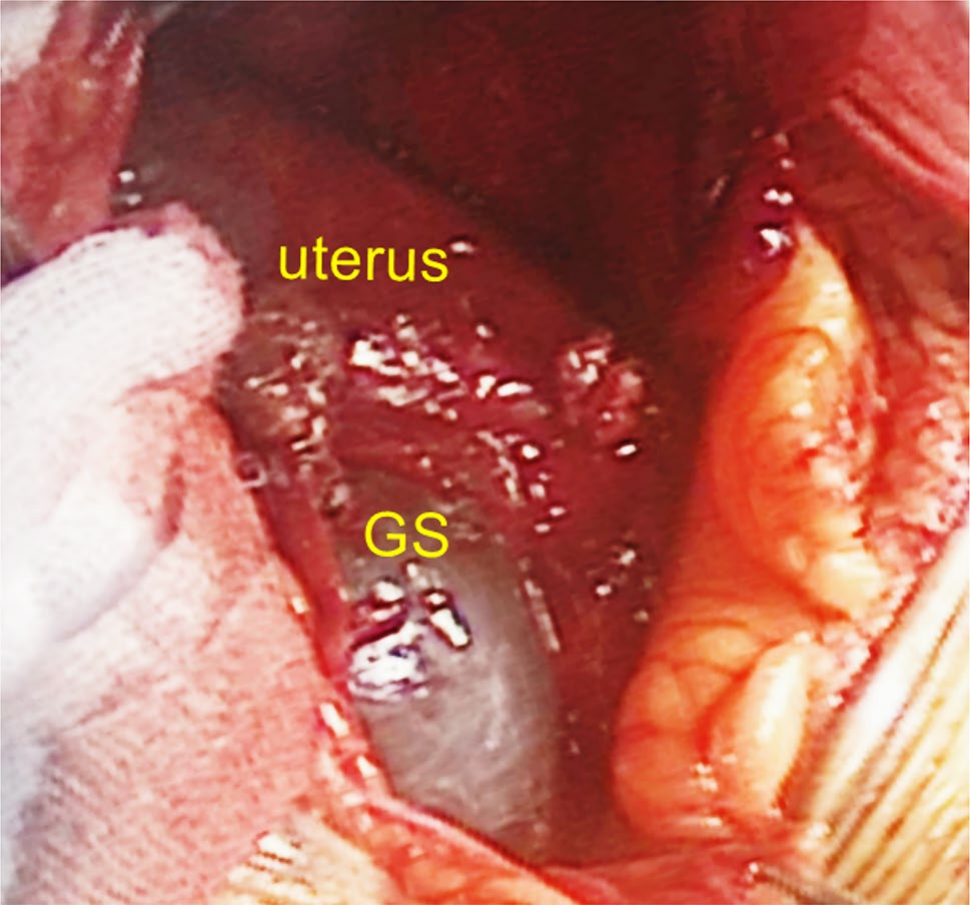
Abdominal pregnancy as seen during the operation. The gestational sac is located behind the uterus

After sufficient preparation, the patient was sent to the operating room for an exploratory laparotomy. During the operation, the gestational sac was seen behind the uterus, with a formed fetus in it, and the amniotic fluid was clear (Fig. 2). The fetus was taken out after ligating the umbilical cord, and the appearance of the fetus had no deformity. After observing for 30 min, except for slight blood oozing from the edge of the placenta, no other evidence of placental abruption was observed. We decided to leave the placenta at site and close the abdomen. The amount of bleeding during surgery was estimated to be 50 ml, and no blood transfusion was required. After the operation, the patient’s body temperature increased to 38.1 ℃, but soon decreased after antibiotic treatment. There was no decrease in hemoglobin levels on the 1st, 3rd, and 8th day after the operation. On the 10th day after the operation, ultrasound-revealed medium echo at the back of the uterus was approximately 7.8 cm long and 4.5 cm thick. There were scattered small echoes but no obvious blood flow signals within. The patient was discharged from the hospital and followed up regularly at the outpatient clinic. The blood β-HCG returned to normal on the 26th day after the operation, but ultrasound still showed a moderate echo of 6.2 × 5.2 × 4.3 cm behind the uterus. The patient has no discomfort and is still under close follow-up.

## Case series

### Methods

We reviewed the cases of abdominal pregnancy that were treated at our hospital and confirmed by intraoperative findings and pathological results. That is, all the cases with “abdominal pregnancy” in the discharge diagnosis were searched in the medical record retrieval system, and the cases with unclear diagnosis and repeated hospitalization were excluded. A total of 17 cases including the current case have been treated at our hospital, and the pregnancy history, diagnosis, treatment, and complications of these patients were analyzed, with an aim to achieve a reference for this case and other similar cases.

### Results

#### Basic information

**Fig. 3:**
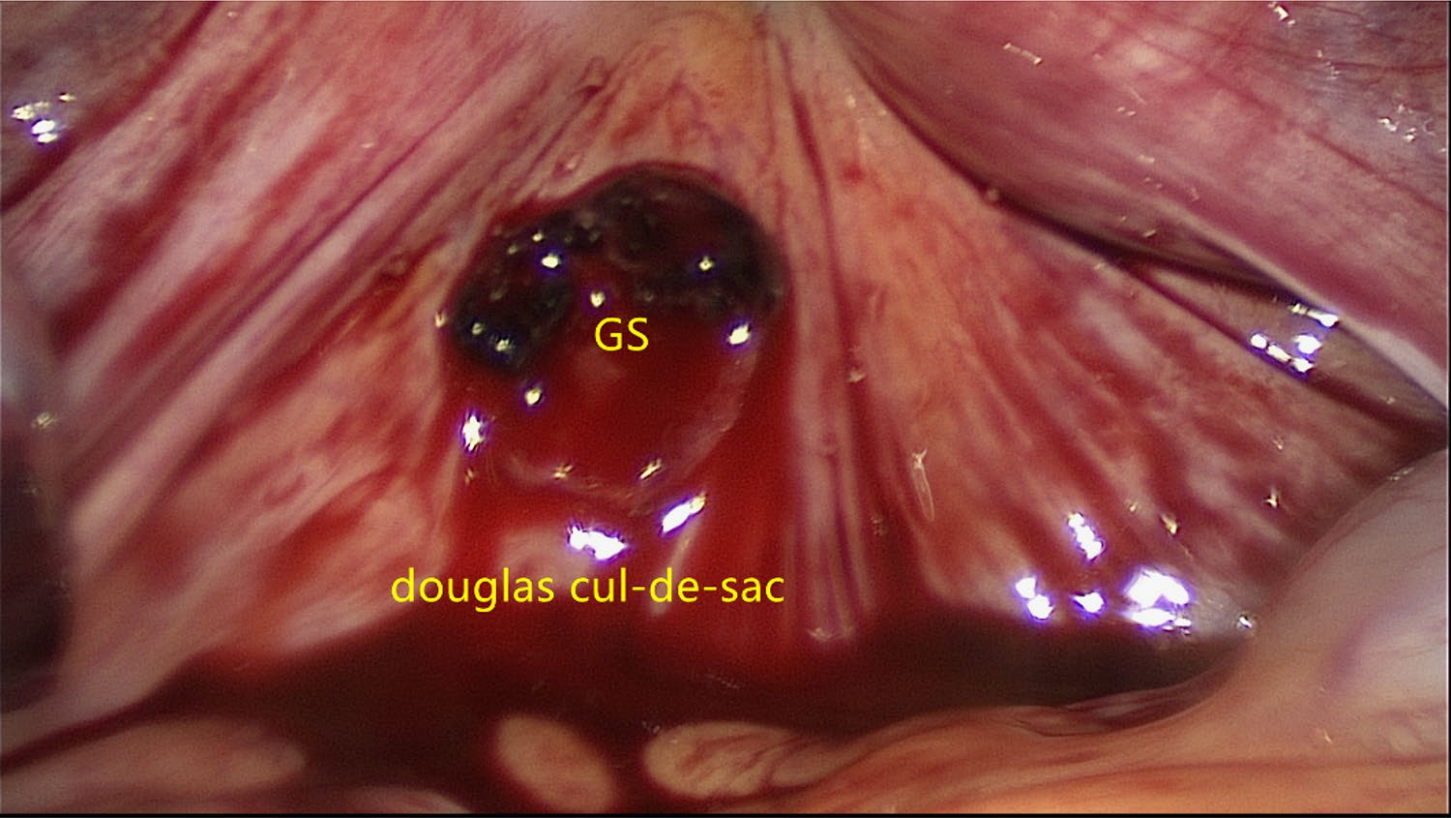
During the operation in CASE 13, the pregnancy sac was found to be located in the peritoneum inside the uterosacral ligament

**Fig. 4:**
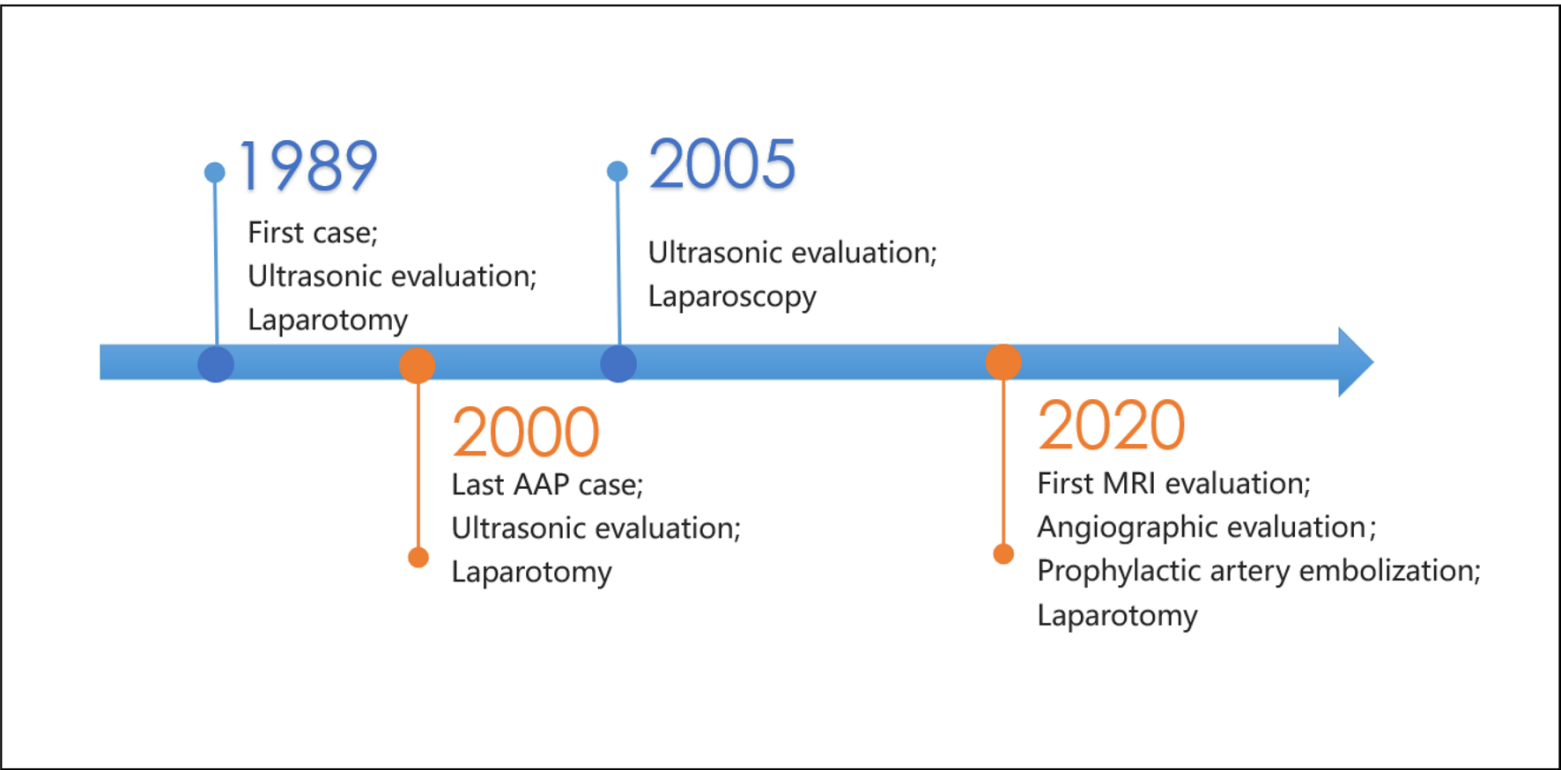
Timeline of diagnosis and treatment procedures for all 17 cases

All 17 cases occurred between 1989 and 2021, in this period, the total number of deliveries and ectopic pregnancies diagnosed in our unit were 62,121 and 9095, respectively. Abdominal pregnancies accounts for approximately 0.19% of all ectopic pregnancies. Among all 17 abdominal pregnancy patients, the median age was 34 years (22–42 years), and the median gestation was 57 days (41 days to 32 weeks). All patients had symptoms, and 15 of 17 (88.24%) had abdominal pain; 7 of 17 (41.18%) patients had vaginal bleeding. The implantation sites of the gestational sac were bladder peritoneal reflection, anterior rectal wall, greater omentum, uterine serosa, medial uterosacral ligament, or uterosacral ligament (Fig. 3). Further, 29.41% (5/17) of patients were diagnosed before surgery, and the other 70.59% (12/17) were misdiagnosed with tubal pregnancy or unexplained intra-abdominal hemorrhage (patients in early pregnancy) and central placenta previa (patients in third trimester) before surgery. The primary method of preoperative evaluation was ultrasound, and it was not until 2020 that MRI and angiography were first added to preoperative diagnostic procedures for suspected abdominal pregnancies (Fig. 4). All 17 patients were treated by laparoscopy or open surgery, no conservative/non-surgical management. Subsequently, 58.82% (10/17) recovered without complications after treatment, 29.41% (5/17) developed fever, which improved after antibiotic treatment, 5.88% (1/17) developed intra-abdominal hemorrhage and hemorrhagic shock, and had to undergo a reoperation, and 5.88% (1/17) had venous thrombosis in both lower extremities and needed additional anticoagulation therapy. No deaths occurred (Table 1).

**Table 1:** Clinical data of 17 cases of abdominal pregnancy

Serial number	Age (years)	Maternity history	Gestational age	Surgery/operation time	Risk factors	abdominal pain (Y or N)	Vaginal bleeding (Y or N)	Preoperative diagnosis	Gestational sac implantation site	Current treatment	Complications	need for blood transfusion (Y or N)	Last follow-up status
1	31	G1P0	Over 5 months	1989/1/6	None	Y	N	Abdominal pregnancy	Right posterior end of the uterus	Laparotomy for fetus retrieval, because of intraoperative bleeding; multiple placenta remains in place	Fever and abdominal pain improved after antibiotic therapy	Y	Underwent another laparotomy 26 months later because of another ectopic pregnancy rupture in the left fallopian tube, and the remaining placenta was removed during this operation. After 41 months, one child was naturally conceived and delivered
2	22	G1P0	27 weeks	1992/5/19	History of induced abortion during first trimester	Y	N	Central placenta previa	The posterior abdominal wall; the lower right is connected to the right appendage. The upper boundary reaches the greater omentum of the mesenteric membrane, and the left side is attached to the left side of the abdominal wall	Laparotomy; the placenta was left in site, and the fetus was malformed	Fever and abdominal pain improved after antibiotic therapy	N	Unknown
3	33	G1P1	32 weeks	1994/4/16	Primary infertility for 4 years with an unknown cause	Y	N	Abdominal pregnancy	Right rudimentary uterus	Laparotomy; the placenta was left in site, intra-abdominal hemorrhage and hemorrhagic shock on the same day; thus, another surgical treatment was performed. The placenta and the residual uterine horns were removed during the second operation. The fetus survived	Intra-abdominal hemorrhage and hemorrhagic shock on the same day	Y	Unknown
4	38	G1P0	23 weeks	2000/4/7	None	Y	Y	Abdominal pregnancy	Left fallopian tube	Left oviduct resection + intra-abdominal fetal removal + uterine fibroid removal + curettage; fetal death preoperatively	Fever improved after antibiotic treatment	Y	Unknown
5	37	G1P0	15 weeks	2004/6/22	Primary infertility, ovulation induction to conceive	Y	N	Abdominal pregnancy	Right fallopian tube	Right oviduct resection + abdominal pregnancy resection, complete removal of placenta and umbilical cord	Venous thrombosis in both lower extremities, which improved after anticoagulant treatment	N	Unknown
6	40	G4P2	3 months	2005/7/18	Before this treatment, due to "right tubal ampullary pregnancy", patient underwent diagnostic curettage and laparoscopic double tube resection, which was confirmed pathologically after the operation	Y	Y	Ectopic pregnancy rupture OrChoriocarcinoma	Right bladder peritoneal reflection	Complete removal of gestational tissues laparoscopically	–	Y	One week after surgery, serum HCG dropped to nearly normal
7	28	G0P0	47 days	2012/11/14	None	Y	N	Ectopic pregnancy	Serous membrane of right anterior wall of the rectum	Complete removal of gestational tissues laparoscopically	–	N	After 12 days, the serum HCG dropped to nearly normal
8	32	G4P0	2 months	2014/3/4	Before this treatment, due to “right tubal ampullary pregnancy”, laparoscopic right salpingectomy was performed, which was confirmed pathologically after the operation	Y	Y	The cause of intra-abdominal bleeding remains to be investigated	Omentum	Laparoscopic exploration + laparotomy and complete resection of part of the greater omentum and lesions	Fever improved after antibiotic treatment	Y	One week after surgery, serum HCG dropped to nearly normal
9	35	G4P1	54 days	2014/12/03	None	Y	N	Ectopic pregnancy	The surface of the right uterine horn	Complete removal of pregnancy tissue on the surface of uterine lesions under laparoscopy	–	N	Three weeks after surgery, serum HCG dropped to normal
10	42	G2P1	51 days	2015/4/10	None	Y	N	Ectopic pregnancy rupture	Medial rectal surface of left uterosacral ligament	Laparoscopic exploration + gestational tissue removal + bowel repair + D&C	–	Y	One month after surgery, serum HCG dropped to normal
11	28	G1P0	57 days	2017/6/6	Before this treatment, due to “right tubal ampullary pregnancy”, laparoscopic right oviduct fenestration of embryos + right fallopian cyst removal was performed, which was confirmed by pathology after surgery	Y	Y	Abdominal pain unexplained Intra-abdominal bleeding	Colonic splenic flexure	Laparoscopy + laparotomy exploration + mesenteric hematoma removal + mesenteric repair, MTX 75 mg intramuscular injection on the 1st and 7th day after the operation	–	Y	One month after surgery, serum HCG dropped to normal
12	40	G2P0	7 weeks	2018/2/19	Bilateral fallopian cysts	Y	N	Left fallopian tube pregnancy	Inner side of left sacroiliac ligament	Laparoscopic exploration + complete removal of the ectopic pregnancy tissues	–	N	After 17 days, serum HCG dropped to normal
13	33	G5P1	48 days	2018/7/24	Pelvic adhesions, history of surgery for ectopic pregnancy in the left fallopian tube	Y	Y	Ectopic pregnancy	Left uterosacral ligament	Laparoscopic exploration + complete removal of the ectopic pregnancy tissues	–	N	After 2 weeks, serum HCG dropped to normal
14	35	G2P0	41 days	2018/8/24	Right fallopian cyst, history of appendectomy	Y	N	Ectopic pregnancy	Serosal surface of the right side of the uterine wall	Laparoscopic exploration + complete removal of the ectopic pregnancy tissues	–	N	After 2 weeks, serum HCG decreased by 50 times and was close to normal
15	34	G3P1	9 weeks	2020/5/5	Pelvic adhesions	N	Y	Left fallopian tube pregnancy	External iliac artery surface of left retroperitoneum	Laparoscopic exploration + left posterior ectopic pregnancy tissue removal + left fallopian tube resection + diagnostic curettage	–	N	Unknown
16	39	G7P3	Over 3 months	2020/12/4	Before this treatment, a medical abortion was performed at the primary hospital after uterine evacuation. Villi were seen during the operation, the details of the past medical record are unknown	N	Y	Abdominal pregnancy	Right anterior of the posterior iliac vessels of the uterus; left uterine artery and left ovarian artery	Placental artery embolization before operation, laparotomic exploration + fetal removal, the placenta remains in site	Fever improved after antibiotic treatment	N	Serum β-HCG returned to normal on the 26th day after the operation, but ultrasound still showed a moderate echo of 6.2 × 5.2 × 4.3 cm behind the uterus
17	30	G3P0	7 weeks	2020/12/13	None	Y	N	Ectopic pregnancy	Left mesentery of the rectum	Laparoscopic exploration + removal of ectopic pregnancy lesions on the surface of the mesentery + diagnostic curettage	–	N	After 20 days, serum HCG dropped to normal

#### Non-advanced abdominal pregnancy

##### Diagnosis and treatment

The gestational age of 13 of 17 (76.47%) patients was less than 20 weeks; of these 2 patients were diagnosed before treatment, and both were in the second trimester and the fetal heartbeat could be seen on imaging. Both these patients underwent laparotomy. Among these two, one patient developed venous thrombosis in both lower extremities after operation. Among the remaining 11 cases that were not diagnosed before operation, three were presumed to be secondary to a recent tubal ectopic pregnancy, although the abnormal tube was removed in two cases during a previous operation; the remaining 8 were primary cases. In addition, all 11 patients in the first trimester were not diagnosed by imaging examinations before surgery; 10 of them had abdominal pain, but 7 of these 10 (70%) were misdiagnosed with tubal pregnancy; four patients received blood transfusion because of gestational sac implanting site rupture and suffered heavy bleeding. The pregnancy tissue was successfully removed in all 11 patients through laparoscopic surgery, and blood β-HCG levels fell to the normal range within 1 month after the operation (Table 2).

**Table 2:** Case characteristics of non-late abdominal pregnancy

Groups	Total cases	Treatment method	Preoperative diagnosis		Abdominal pain		Need for blood transfusion	
			Cases	Percentage (%)	Cases	Percentage (%)	Cases	Percentage (%)
First trimester	11	Laparoscopic surgery	0	0	10	90.91	4	36.36
Second trimester	2	Laparotomy	2	100	1	50	0	0

##### Risk factors

In all, 9 of 13 (69.23%) patients with a gestational age of less than 20 weeks had high risk factors for an abdominal pregnancy: 1 patient was fertilized by ovulation induction because of primary infertility; 2 had intrapelvic adhesions; 2 had cysts in the fallopian tubes; 2 had a history of pelvic surgery, particularly fallopian tube surgery; 1 had a history of uterine cavity operation in early gestation and 3 had a history of tubal ectopic pregnancy confirmed by laparoscopy and pathology.

#### Advanced abdominal pregnancy

##### Diagnosis and treatment

Overall, 23.53% (4/17) of patients had AAP, that is, gestational age > 20 weeks. All four cases were observed in 2000s and before. All four patients had symptoms of abdominal pain, three were diagnosed by imaging examinations before surgery, and all received blood transfusions because of excessive bleeding during the perioperative period. Among the four patients, there was one fetal malformation, one stillbirth, and one live birth. With regard to the placenta, in two cases, the placenta was attached to a fallopian tube and ruptured uterus with remnant uterine horn, respectively. This is speculated to be secondary to tubal pregnancy and remnant uterine horn pregnancy. The four patients were treated via laparotomy. In one patient, the placenta attached to the left fallopian tube was removed during the operation, and the placenta was left in site in three other cases. However, one patient showed intra-abdominal hemorrhage and hemorrhagic shock on the same day; thus, the patient needed another surgical treatment. During the second operation, it was found that the placenta remained in situ attached to a ruptured residual uterine horn, which was not accurately recognized by imaging before and during the first operation. Another patient underwent another laparotomy 26 months later because of another ectopic pregnancy ruptured in the left fallopian tube, and the remaining placenta was removed during this operation. All four patients developed fever after operation, but antibiotic treatment was effective, and they were discharged after a good recovery.

##### Risk factors

Overall, 50% (2/4) of patients had high risk factors for abdominal pregnancy in this patient group. Among the two patients, one had a history of uterine cavity operation in early pregnancy and the other was naturally conceived after four years of infertility.

## Discussion

In the past 22 years, the ratio of ectopic pregnancy to delivery numbers in our hospital was about 14.64% (9095/62121), which is higher than that reported in other literatures. This may be because our hospital is Chinese National Clinical Research Center for Obstetrics & Gynecologic Diseases, and accepts referral patients with difficult and severe diseases in obstetrics and gynecology from Chinese capital and surrounding areas, including rare ectopic pregnancies such as cesarean scar pregnancy, cervical pregnancy, etc. In addition, some patients suspected of ectopic pregnancy in other hospitals hope to receive further diagnosis and treatment in our hospital and voluntarily request referral to our hospital. Therefore, these data are not representative of the level of ectopic pregnancy in the region over the 22-year period.

An abdominal pregnancy can be primary (wherein the blastocyst is directly implanted on the surface of the peritoneum or the viscera of the abdominal cavity) or secondary (wherein the embryo falls from the fallopian tube into the abdominal cavity). In our study, there are three cases of tubal ectopic pregnancy that occurred not long ago. Two cases of AAP in this study, where the placenta was attached to a fallopian tube/ruptured residual uterine horn, could be considered secondary or uterine cavity manipulation in the first trimester (CASE 2 and CASE 16), which may cause iatrogenic perforation of the uterus and free the gestational sac into the abdominal cavity [8]; however, this cannot be confirmed. The diagnostic criteria for primary abdominal pregnancy have been proposed by Studdiford [9] in 1942: (1) both fallopian tubes and ovaries are normal; (2) no uterine-peritoneal fistula formation; (3) Pregnancy only exists in the abdominal cavity; and (4) there is no possibility of tubal pregnancy. Ten of our cases can be considered primary according to this standard. Although we consider the diagnostic criteria to be ambiguous in the definition of “no possibility of tubal pregnancy”, since in our case, most abdominal pregnancies were implanted in the pelvic cavity and both fallopian tubes and ovaries were normal. But whether they had a tubal pregnancy followed by a complete miscarriage into the pelvic cavity was not identifiable. Our recommendation is that when β-HCG is elevated and the gestational sac cannot be located intrauterine, ultrasound or MRI should be attempted first in the pelvic cavity, including the fallopian tube and surrounding area, regardless of whether there had been a tubal pregnancy.

The risk factors of abdominal pregnancy include fallopian tube injury, pelvic inflammatory disease, endometriosis, and pluripara among others [10]. In a literature review of the case reports of abdominal pregnancy after in vitro fertilization-embryo transfer [11], 37% of cases of abdominal pregnancy have a history of tubal ectopic pregnancy, and 61% of cases have anatomical/structural infertility, with fallopian tube factors being the most common; the incidence in fresh embryo transfer (71%) is much higher than that in frozen embryo transfer (11%). In addition, there are also reports that the use of cocaine may be a risk factor for abdominal pregnancy [12]; further, the incidence in non-industrialized countries is higher than that in industrialized countries [13].

The clinical symptoms of an abdominal pregnancy are uncertain. According to our research, most patients have abdominal pain and vaginal bleeding [14]. In AAP, abdominal pain may manifest as fetal movement pain [15]; other symptoms include placenta and fetal position abnormalities, abnormal cervix position, and failure to induce labor [16]; some patients may be admitted for shock cause by rupture of ectopic pregnancy lesions. Excessively elevated alpha-fetoprotein levels in laboratory tests can also be used to guide diagnosis [17]. Imaging examinations such as ultrasound, MRI, and computed tomography (CT) are essential in confirming the diagnosis, and can be used to evaluate the position of the gestational sac, blood supplies, implantation site, and bleeding lesions [18]; even in our treatment experience, vascular ultrasound and angiography could also be used to evaluate the blood supply of the placenta. Only 50% of early abdominal pregnancy can be diagnosed by ultrasound [19], but when combined with serum β-HCG levels, the sensitivity of ultrasound increases. MRI, as a multi-planar, multi-parameter imaging method with high resolution of soft tissues and no radiation, can accurately visualize the intra-abdominal structure, can show the anatomical relationship among the fetus, placenta, and the maternal organs in detail, and can also show vascular invasion [20]. Although the safety of MRI plain scan during pregnancy has long been affirmed [21], the intravenous gadolinium contrast agent used in enhanced MRI is listed in the US Food and Drug Administration (FDA) drug classification Class C pregnancy drugs, and studies have shown that intrauterine gadolinium exposure is related to stillbirths, neonatal deaths, and various skin abnormalities [22]; therefore, when there is a possible intrauterine pregnancy or AAP wherein the fetus is expected to be preserved, enhanced MRI scans should be performed with caution. Although after undergoing imaging evaluations mentioned above, abdominal pregnancy may be diagnosed and treated early in areas with well-developed medical conditions, in areas with insufficient medical resources and inadequate prenatal care, abdominal pregnancy may be diagnosed at greater gestational age or even full-term [23]. In our case, all AAPs occurred in 2000 and before, which is consistent with the rapid development of our country.

Of the 17 cases of abdominal pregnancy in our study, only five (29.41%) were diagnosed before surgery, and the accuracy of diagnosis increased with the increase in gestational age and the appearance of fetal heart rate. Further, seven (41.18%) of the total patients required blood transfusion during the perioperative period; this percentage among AAP cases was 75%. All our patients were treated surgically. There are no standard treatment methods acknowledged, and no predictive standards for successful medical management [24]. Previous literature reports on the therapeutic regimen include conservative treatments and surgical treatments. Conservative treatments include selective placental vascular embolization, ultrasound-guided drug injection in the gestational sac, or maternal systemic drug therapy [25, 26]. Conservative treatments may need a long follow-up period. A previous study reported that a 14 week gestation was terminated by ultrasound-guided induction, and the fetus and placenta remained in site. The follow-up visit revealed that the gestational sac degraded very slowly, and only a small amount amniotic fluid volume was reduced at 9 months after surgery [27].

Surgical treatment is the most common treatment for abdominal pregnancy. Although laparotomy has an irreplaceable advantage over laparoscopic surgery in terms of rapid and adequate hemostasis, there are still many reports on laparoscopic surgery for the treatment of abdominal pregnancy [28], especially in early stages of the pregnancy, laparoscopic removal the ectopic pregnancy tissues can be tried [29], which achieved an excellent therapeutic effect in our case. However, before the operation, the site of implantation of the gestational sac should be fully assessed via imaging as much as possible, and a multidisciplinary team should cooperate to prepare for hemostasis and salvage, and to choose selective embolization, if necessary.

Abdominal pregnancies in the second and third trimesters were all treated through laparotomy, but because of the fear of massive bleeding after placental dissection [30] and maternal perinatal death [31]; further, in 4 (66.67%) of the six cases with the second and third trimester abdominal pregnancy, it was chosen to keep the placenta at the site. However, it is recommended to fully evaluate the position and blood supply of the placenta, because when medical care was inadequate, we had insufficient preoperative evaluation. This led to massive intra-abdominal hemorrhage and patient went in a hemorrhagic shock a few hours after the first laparotomy; the second operation proved that the placenta was attached to the ruptured rudimentary uterus and was partially abrupted. In this case, the placenta was resectable during the first operation. In addition, in the latest case, we tried to determine the blood supply to the placenta through a vascular ultrasound and angiography before surgery, and then embolized the placental blood vessel [32], which significantly reduced perioperative bleeding and avoided the need of a blood transfusion. In the case where the placenta was left in site, we chose to stop administering drugs and waited for self-absorption to avoid the use powerful drugs to cause rapid necrosis of the placenta, which can cause severe intra-abdominal infection [33]. However, in previous literature reports, some physicians believed that post-operative residual placenta still needs drug treatment, and tried to apply small-dose methotrexate systemic therapy for some time after surgery [34], which can also avoid complications such as infection and bleeding. Further, this same physician, after collecting relevant literature from the database and relevant data on the case, proposed that when the gestational sac is planted in a vascular-rich area such as the iliac vascular area, even if the residual placenta has no blood flow, the nearby blood vessels could be torn because of activity, causing massive bleeding [35]. Accordingly, he proposed that when the patient is stable and the placental blood flow stops approximately 3 months after the termination of pregnancy, the placenta should be surgically removed again to avoid the risk of further bleeding and infection. However, in our cases, the placenta left in site was followed up for a maximum period of 26 months, and during this period, the condition of patients was stable.

Interestingly, there is a striking similarity with the intrauterine placenta accreta spectrum cases and abdominal pregnancy, especially in the placenta management of AAP. Intentional Retention of the placenta (IRP) [36] is intentionally leaving placenta in the uterus after delivering the baby, including subsequent removal/non-removal of the placenta. Similar to AAP, the placenta accreta spectrum may be associated with invasion and damage to other organs. In addition, fertility preservation needs to be considered, as some women may wish for subsequent pregnancies. Some physicians believe that IRP is a reasonable option to reduce the risk of catastrophic bleeding because it avoids abundant blood supply of periuterine during the postpartum period [37], but other reports believe that it increases the rate of arterial embolism, infection, and the risk of re-hospitalization [38]. The difference in the treatment of AAP in placenta is that the risk of infection after placenta retention is lower than that of IRP due to the lack of direct communication with the outside world. However, with the lack of direct communication with the outside world, the placenta remains in the abdominal cavity, fatal hemorrhage is initially undetectable, and the patient may not be noticed until hemorrhagic shock.

According to reports in the literature, an abdominal pregnancy has a higher incidence of fetal malformations and perinatal mortality [6]. Among our four patients with AAP, there was one case of fetal malformation and one stillbirth, and only one live birth. Previous studies have analyzed literature reports of 39 cases of abdominal pregnancy, of which only two cases reported neonatal survival [39]. This may be related to the unstable blood supply to the placenta in the abdominal cavity and fetal stress deformity [40].

Abdominal pregnancy is a rare ectopic pregnancy. Although most abdominal pregnancies can be detected and terminated at an early stage with the popularization of prenatal care, its diagnosis and treatment are still a big challenge in areas with insufficient medical resources. In addition, for abdominal pregnancies in the second and third trimesters, especially AAP, conservative treatment methods and the treatment of forced in site placenta also need to be further supported and explored by evidence-based medical studies.
